# miR-let-7a inhibits sympathetic nerve remodeling after myocardial infarction by downregulating the expression of nerve growth factor

**DOI:** 10.1515/med-2024-0975

**Published:** 2024-06-12

**Authors:** Yanyan Jing, Lei Qi, Xueli Zhang, Lu Zheng, Peijin Yang, Jie Yin, Yugen Shi, Suhua Yan

**Affiliations:** Department of Cardiology, Shandong Qianfoshan Hospital, Cheeloo College of Medicine, Shandong University, Jinan, China; Department of Cardiology, Yantai Yuhuangding Hospital, Shandong University, Yantai, China; Shandong First Medical University & Shandong Academy of Medical Sciences, Jinan, China; Department of Cardiology, The First Affiliated Hospital of Shandong First Medical University & Shandong Provincial Qianfoshan Hospital, Shandong Medicine and Health Key Laboratory of Cardiac Electrophysiology and Arrhythmia, Shandong, 250014, China; Department of Cardiology, The First Affiliated Hospital of Shandong First Medical University & Shandong Provincial Qianfoshan Hospital, Shandong Medicine and Health Key Laboratory of Cardiac Electrophysiology and Arrhythmia, 16766 Jingshi Road, Jinan, Shandong, 250014, China

**Keywords:** miR-let-7a, nerve growth factor, macrophages, sympathetic neural remodeling, myocardial infarction

## Abstract

**Objective:**

Sympathetic hyperinnervation following myocardial infarction (MI) is one of the primary causes of ventricular arrhythmias (VAs) after MI. Nerve growth factor (NGF) is a key molecule that induces sympathetic nerve remodeling. Previous studies have confirmed that microRNA (miR)-let-7a interacts with NGF. However, whether miR-let-7a is involved in sympathetic remodeling after MI remains unknown. We aimed to investigate whether miR-let-7a was associated with the occurrence of VA after MI.

**Methods and results:**

A rat model of myocardial infarction was established using left coronary artery ligation. miR-let-7a expression levels were analyzed by reverse transcription-quantitative PCR. Western blotting was also used to examine NGF expression levels *in vivo* and in M1 macrophages *in vitro*. The relationship between miR-let-7a and NGF levels was investigated using a luciferase reporter assay. The results revealed that the expression of miR-let-7a decreased significantly after MI, while NGF expression was significantly upregulated. In addition, overexpression of miR-let-7a effectively inhibited NGF expression in rats, which was also verified in M1 macrophages. Tyrosine hydroxylase and growth-associated protein 43 immunofluorescence results revealed that the administration of a miR-let-7a overexpression lentivirus to rats inhibited sympathetic remodeling after MI. Programmed electrical stimulation, renal sympathetic nerve activity recording, and heart rate variability measurements showed that miR-let-7a overexpression decreased sympathetic activity.

**Conclusions:**

These findings provide novel insights into the molecular mechanisms by which miR-let-7a and NGF contribute to the progression of sympathetic nerve remodeling after MI. Therefore, miR-let-7a may be a promising therapeutic target to reduce the incidence of arrhythmia following MI.

## 1 Introduction

Myocardial infarction (MI) is a severe cardiovascular disease [1]. Following MI, the risks of ventricular tachyarrhythmias (VA) and sudden cardiac death increase. It has previously been demonstrated that sympathetic hyperactivity can affect cardiac electrophysiological activity, and an increase in sympathetic nerve density is one of the main causes of VA and sudden cardiac death [2,3]. Numerous researchers have studied the mechanisms of sympathetic remodeling following MI [4,5,6]. Studies have found that sympathetic nerve regeneration and remodeling are closely related to the inflammatory response, mainly in the area around the infarction, where inflammatory cells and inflammatory factors accumulate in large quantities. Macrophages play a key role in sympathetic nerve remodeling by secreting nerve growth factor (NGF) [7,8]. Our previous study also confirmed that NGF secretion promoted the occurrence of VA after MI [9,10]. However, the mechanism by which NGF is regulated requires further investigation.

MicroRNAs (miRNAs/miRs) are short strands of RNA that are 20–23 nucleotides in length and are widely expressed in eukaryotes. miRNAs can bind to the 3′-untranslated regions (UTRs) of mRNAs, thereby inhibiting protein translation and inducing target mRNA degradation [11]. Notably, the miR-let-7 family was one of the first miRNA families to be identified, and this family includes 12 highly conserved subtypes including miR-let-7a. This miRNA principally exerts anti-inflammatory effects by inhibiting specific genes that target downstream signaling pathways [12]. It has been documented that miR-let-7a participates in the process of heart failure by regulating the activity of the β1-adrenoceptor [13]. In addition, miR-let-7a has been shown to directly inhibit cardiac hypertrophy by regulating calmodulin [14]. Although evidence suggests the importance of miR-let-7a in cardiovascular diseases, its role in arrhythmias remains unknown, and further investigation is required.

Therefore, the present study aimed to explore the mechanism by which miR-let-7a participates in the occurrence and development of VA following MI, and the association between miR-let-7a and NGF. This study is intended to generate new ideas for future research investigating the mechanism of VA after MI and may suggest a new direction for targeted clinical therapies.

## 2 Materials and methods

### 2.1 Animal and MI model

Male Sprague-Dawley rats, aged 8 weeks and weighing 260 ± 20 g, were provided by Beijing Vital River Company. The rats were housed in a standard animal room at a temperature of 21 ± 1°C under a 14/10 h light/dark cycle with free access to water and food.

**Figure A1:**
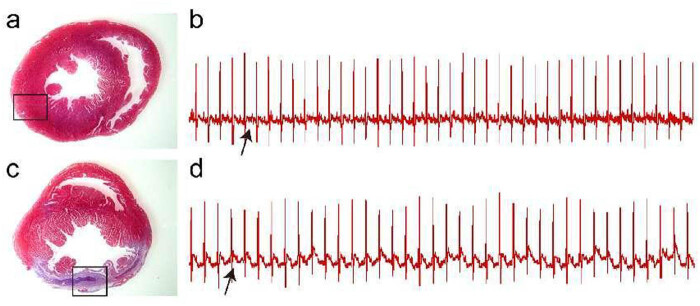
The success of the MI model. (a) and (b) Representative image of hearts stained with Masson’s Trichrome and electrocardiogram image in the sham groups. (c) and (d) Representative image of hearts stained with Masson’s Trichrome and electrocardiogram image in the MI groups. MI: Myocardial infarction.

The rats were anesthetized with intraperitoneally administered 3% pentobarbital sodium (30 mg/kg). Following tracheotomy, respiratory support was provided to the rats using a small animal ventilator (TME Technology Company, Chengdu, China) at a rate of 30–40 breaths/min and a tidal volume of 1.1–1.3 ml/100 g. The chest cavity was opened along the left axilla to expose the heart, and the left coronary artery was ligated 2–3 mm from its origin in the pulmonary artery. The ligation material was 6–0 polypropylene thread. The successful establishment of MI was easily identified by local cyanosis of the myocardium, weakened ventricular wall motion, and ST-segment elevation or VAs [ventricular tachycardia (VT) or ventricular fibrillation], as shown by electrocardiography (Figure A1). To gain a clear understanding of the clinical manifestations, only rats with infarctions ranging from 30 to 50% were assigned to the MI group. In the sham group, the heart was exposed but the artery was not ligated.


**Ethical approval:** This study was approved by the Ethics Committee of The First Affiliated Hospital of Shandong First Medical University & Shandong Provincial Qianfoshan Hospital. All procedures were performed following the Guidance Suggestions for the Care and Use of Laboratory Animals, formulated by the Ministry of Science and Technology of China.

### 2.2 Cell preparation

**Figure A2:**
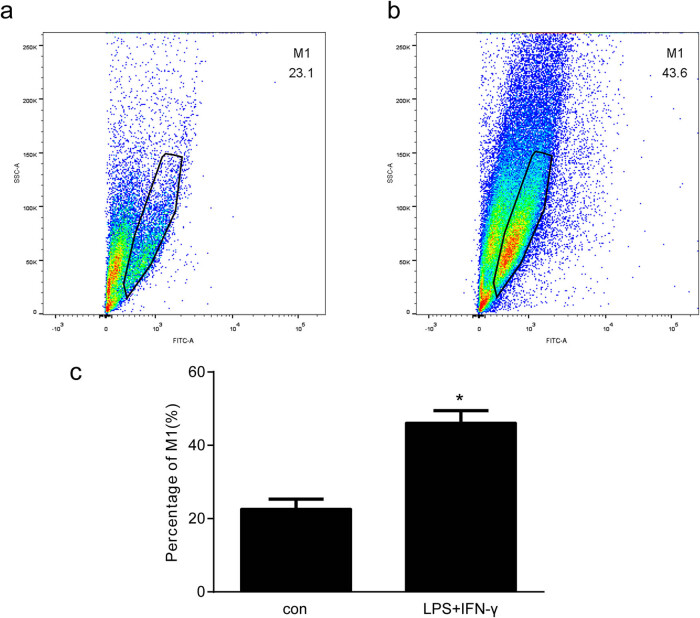
(a) Flow cytometry image of M0 macrophages. (b) Flow cytometry image of M1 macrophages. (c) The percentage of M0 macrophages that had been converted into M1 macrophages. Data are presented as the mean ± SD. ^*^ *p* < 0.05 vs con. SD: standard deviation.

The rat macrophages (NR8383) were purchased from Wuhan Procell Life Science &Technology Co., Ltd. According to our previous study [15], M1 macrophages are cells that play a major proinflammatory role in the early stages of acute MI. Cells were stimulated with 10 μg/ml lipopolysaccharide (LPS) (Sigma-Aldrich, Shanghai, China) and 0.05 μg/ml interferon (IFN)-γ (Shanghai Academy of Sciences) for 24 h at 37°C in humidified air with 5% CO_**2**_ to induce the transformation of M0 macrophages into M1 macrophages (Figure A2).

### 2.3 Experimental design

Three separate experiments were conducted.

#### 2.3.1 Protocol 1

Fifty rats were divided into five groups (*n* = 10 per group): sham and MI after 1, 3, 5, and 7 days. The expression of miR-let-7a was detected using reverse transcription-quantitative polymerase chain reaction (RT-qPCR) and western blotting. The temporal expression of NGF was measured using RT-qPCR and western blotting.

#### 2.3.2 Protocol 2

M0 macrophages were induced to become M1 macrophages as described above. M1 macrophages were divided into two groups: M1 + negative control (NC) and M1 + Len-let-7a. The M1 + Len-let-7a group was transduced with 50 nM miR-let-7a-overexpressing virus and the M1 + NC group was transduced with 50 nM of the control virus. The viruses were purchased from Genomeditech (Shanghai, China). Protein and RNA were extracted from the cells 48 h after experimentation at 37°C to observe changes in NGF and miR-let-7a.

#### 2.3.3 Protocol 3

**Figure A3:**
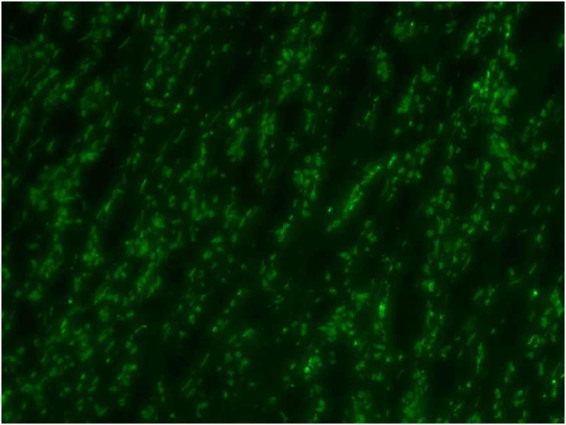
Fluorescent microscopic image showing GFP expression, indicating that the lentivirus could be efficiently delivered in vivo via heart injection after 1 week.

Seventy rats were divided into four groups: sham + NC (*n* = 15), sham + Len-let-7a (*n* = 15), MI + NC, and MI + Len-let-7a (*n* = 20 per group). The miR-let-7a-overexpressing lentivirus or control virus was synthesized by Genomeditech. The lentivirus was 1.08 × 10^8^ genomic particles/mL. A total of 150 μL lentivirus was intramyocardially injected at four sites at the infarcted border (>2 mm around the infarcted area) of the left ventricle (LV) at a depth of 1–2 mm with a 30-gauge needle after ligation. Sympathetic hyperinnervation has previously been reported to peak on day 7 in large rodents; therefore, we sacrificed rats 7 days after MI [3]. One week after injection, the lentivirus with the green fluorescent protein (GFP) reporter gene was successfully transfected into the myocardium by immunofluorescence localization of GFP expression (Figure A3). Before sacrifice on day 7 post-MI, heart rate variability (HRV), renal sympathetic nerve activity, and programmed electrical stimulation were measured. The heart was removed for analysis using western blotting, RT-qPCR, ELISA, and immunofluorescence.

### 2.4 HRV measurements

Telemetry recording devices (TR50B, ADInstrument, Australia) were implanted into the abdominal cavity of rats according to a previously reported study [16]. Briefly, one electrode was placed on the xiphoid process on the chest, while the other electrode was fixed on the sternocleidomastoid muscles. Electrocardiogram (ECG) data were continually recorded by a PowerLab physiology system and analyzed using LabChart Pro software (AD Instruments, Australia). Two major frequency components were tested: a high-frequency band (HF; 0.75–2.5 Hz) and a low-frequency band (LF; 0.05–0.75 Hz). Then, a 15 min ECG recording was selected to analyze HRV, and the ratio of the LF to HF bands was calculated. Telemetric recordings were conducted for 7 days before the devices were removed.

### 2.5 Renal sympathetic nerve activity recording

Renal sympathetic nerve activity (RSNA) recording was performed as previously described [17]. In brief, the left renal sympathetic nerves were separated using fine glass needles, and the distal terminus of the renal nerve was displaced. The central part of the nerve was placed on a pair of platinum electrodes and the RSNA was then recorded using power (AD Instruments, Australia). Background noise was detected after the renal sympathetic proximal section, and the experimental data baseline was corrected. The baseline level of RSNA was defined as 100% of the absolute value after subtracting the noise level. The data were analyzed using LabChartPro software (AD Instruments, Australia).

### 2.6 Programmed electrical stimulation

Rats were anesthetized and subjected to ventricular programmed electrical stimulation (PES). A specially modified electrode was placed on the surface of the left ventricular epicardium and a stimulation protocol was conducted as described in our previous study [2]. Then, VA was induced under ventricular stimulation with a basic cycle duration of 150 ms (S0), and S1, S2, and S3 external stimuli were administered after eight rhythmic beats. The stimulation protocols were completed within 10 min, and the arrhythmia scoring system was used to analyze the degree of induced VA. These procedures were executed and recorded using an animal biological function experimental system (BL-420S).

### 2.7 Tissue collection

The rats were sacrificed using an overdose of 3% sodium pentobarbital. The infarct area was generally mottled and pale with a thinned wall. The myocardium extending 0.5–1.0 mm from the infarct scar is generally considered to be an infarcted myocardium. The peripheral region of the left ventricular infarction, specifically the myocardium within 3 mm of the infarcted area, was selected for analysis by immunofluorescence staining, western blot analysis, RT-qPCR, and ELISA. One part of this region was embedded in an optimal cutting temperature compound (OCT) and prepared for immunofluorescence. The remaining part was immediately stored at –80 ˚C for biochemical analysis. In addition, whole heart tissue was required for Masson staining to determine infarct size and was subjected to 10% formalin fixation.

### 2.8 Western blotting

The lysis buffer used for the extraction of proteins from cardiac tissues was a mixture of RIPA (Beyotime Biotechnology) and PMSF at a ratio of 100:1. The protein concentration in the extracted lysate was quantified using a BCA kit (Vazyme Biotech, Nanjing, China). Based on the molecular weight of NGF, 12% sodium dodecyl sulfate-polyacrylamide gels were used for electrophoresis of the protein extracts. For transfer, a polyvinylidene fluoride (PVDF) membrane (Millipore, MA, USA) with a pore size of 0.22 μm was used for NGF, while a pore size of 0.45 μm was used for GAPDH. After transferring the proteins to the membranes, the PVDF membranes were blocked for 1 h at 4°C with 5% skimmed milk. Rabbit polyclonal anti-NGF (1:2,500; Abcam, Cambridge, UK) and rabbit monoclonal anti-GAPDH (1:1,000; Cell Signalling Technology, MA, USA) were used to label NGF and GAPDH, respectively, at 4°C overnight. The membranes were then incubated with horseradish peroxidase-conjugated goat anti-rabbit secondary antibodies (1:5,000, Absin, Shanghai, China) at room temperature for 2 h. Protein bands were detected using an ECL chromogenic substrate (Millipore) before being placed in a FluorChem E imager (Protein Simple, USA) for exposure and visualization. For protein extraction, the cells were first centrifuged at 95*g* for 3 min and repeatedly washed with precooled PBS. A mixture of RIPA buffer and PMSF at a ratio of 100:1 was added to the cells for lysis. The lysed cells were placed on ice and centrifuged at 13,586*g* for 25 min at 4°C. The remaining steps were the same as those used for protein extraction from tissues.

### 2.9 Quantitative polymerase chain reaction (qPCR)

RNA was extracted from cardiac tissue or cells using an RNA-easy isolation reagent (Vazyme Biotech). RNA was reverse transcribed into cDNA using a high-capacity cDNA reverse transcription kit (Thermo Fisher Scientific, MA, USA). The miRNA and mRNA levels were detected using a Bio-Rad iQ5 Multicolour Real-Time PCR system (Bio-Rad Laboratories, CA, USA) with SYBR Green (Vazyme Biotech). U6 and GAPDH were used as the internal references for miRNA and NGF, respectively. The expression levels of miR-let-7a and NGF in each sample were analyzed using the 2^−ΔΔCt^ method. RT-qPCR primer sequences (Takara, Beijing, China) used are listed in Table 1.

**Table 1:** Quantitative PCR primer sequences

Names	Primer sequence (5′–3′)
U6	Forward: GGAACGATACAGAGAAGATTAGC
	Reverse: TGGAACGCTTCACGAATTTGCG
miR-let-7a	CGTGAGGTAGTAGGTTGTATAGTT
GAPDH	Reverse: ATCCGTTCACACCGACCTTC
	Forward: TCTCTGCTCCTCCCTGTTCT
NGF	Reverse: CTGTCACACGCGGGCAGCTATT
	Forward: TTTTGATCGGCGTACAGGCA

miR, microRNA; NGF, nerve growth factor.

### 2.10 Flow cytometry

After counting the cells, the cell supernatant was removed and the cells were washed with 0.5% bovine serum albumin (BSA) solution (Servicebio, Wuhan, China). An anti-CD86 antibody (Abcam) was then added, and the samples were incubated at 4°C for 30 min. After incubation, the cells were washed with BSA and incubated with PBS. Finally, the cell phenotypes were determined using a flow cytometer (BD FACSAria II, USA) to verify the percentage of M0 macrophages that had been converted into M1 macrophages, and the data were analyzed using FlowJo 7.6.1 software (FlowJo LLC) (Figure A2).

### 2.11 Dual-luciferase reporter assay

TargetScan analysis (http://www.targetscan.org/vert_72/) was employed to predict possible target genes of let-7a. NGF was identified as a candidate target gene of let-7a, with a seed sequence in the 3′ untranslated region (UTR). The miR-let-7a expression plasmid (GV268) and control GV268 plasmid were purchased from Shanghai GeneChem, Shanghai, China. The wild-type and mutant sequences of the NGF 3′-UTR were custom-synthesized by GeneChem and cloned into the GV272 firefly luciferase plasmid (GeneChem). The miR-let-7a miRNA response element (MRE) in the wild-type NGF sequence was 5′-CUACCUCA-3′ and the miR-let-7a MRE in the mutant sequence was 5′-AGCAACGA-3′. 293T cells purchased from the Type Culture Collection of the Chinese Academy of Sciences at 80% confluence in 24-well plates were transfected using Lipofectamine^®^ 2000 Reagent (Thermo Fisher Scientific, MA, USA), according to the manufacturer’s protocol. The firefly luciferase plasmid and miR-let-7a expression plasmid were co-transfected with the pRL-TK Renilla luciferase vector (Promega) for normalization. At 48 h after transfection, luciferase activity was measured using the Dual-Glo Luciferase Assay System (Promega). Firefly luciferase activity was normalized to the Renilla luciferase activity.

### 2.12 Masson’s trichrome staining

The hearts of the rats in each group were cut into three parts along the upper and lower edges of the infarct area. The middle part, which contained the entire infarcted myocardium, was removed. Specimens were placed in 10% formalin, incubated at 4°C for 24 h, and embedded in paraffin. The prepared paraffin blocks were sliced into 5 μm sections for Masson staining. The infarct size (%) was determined as the average percentage of the left ventricular infarct size and left ventricular peripheral area. Representative images of the hearts stained with Masson’s trichrome are shown in Figure A1.

### 2.13 Enzyme-linked immunosorbent assay (ELISA)

IL-1β and tumor necrosis factor-α (TNF-α) levels were detected using an ELISA kit (Jianglai, Shanghai, China). The optical density (OD) value at 450 nm was used to determine protein concentrations. The myocardial tissue concentrations of IL-1β and TNF-α were recorded in pg/mg.

### 2.14 Immunofluorescence

First, the OCT-embedded tissue was placed into a freezing microtome and cut into 6 µm sections. After fixing the frozen sections with cold acetone at 25°C for 5 min, they were treated overnight at 4°C with sheep polyclonal anti-tyrosine hydroxylase (TH) (1:400, Millipore) and rabbit polyclonal anti-growth-associated protein 43 (GAP43) (1:100, GeneTex, CA, USA). Following incubation with the primary antibody, FITC-conjugated rabbit anti-sheep (1:100, Bethyl Laboratories, Montgomery, TX, USA) or Alexa 546-conjugated donkey anti-rabbit (1:200, Thermo Fisher Scientific) antibodies were added, and the sections were incubated for 2 h at room temperature. The sections were washed and placed under a fluorescence microscope for observation and image capture. Nerve density was measured and evaluated using ImageJ software.

### 2.15 Statistical analysis

Data are presented as mean ± SD. The Shapiro–Wilk method was used to test the Gaussian distribution of the data. Pairwise comparisons were made using Student’s t-test or a non-parametric Mann–Whitney *U* test using SPSS (version 22.0; SPSS, Inc., Chicago, IL, USA) and GraphPad Prism 8.0 software (GraphPad Software, CA, USA). Multiple group analyses were performed using one-way analysis of variance followed by Tukey’s post hoc test. *p* < 0.05 was considered statistically significant.

## 3 Results

### 3.1 miR-let-7a is downregulated and NGF is upregulated after MI

**Figure 1:**
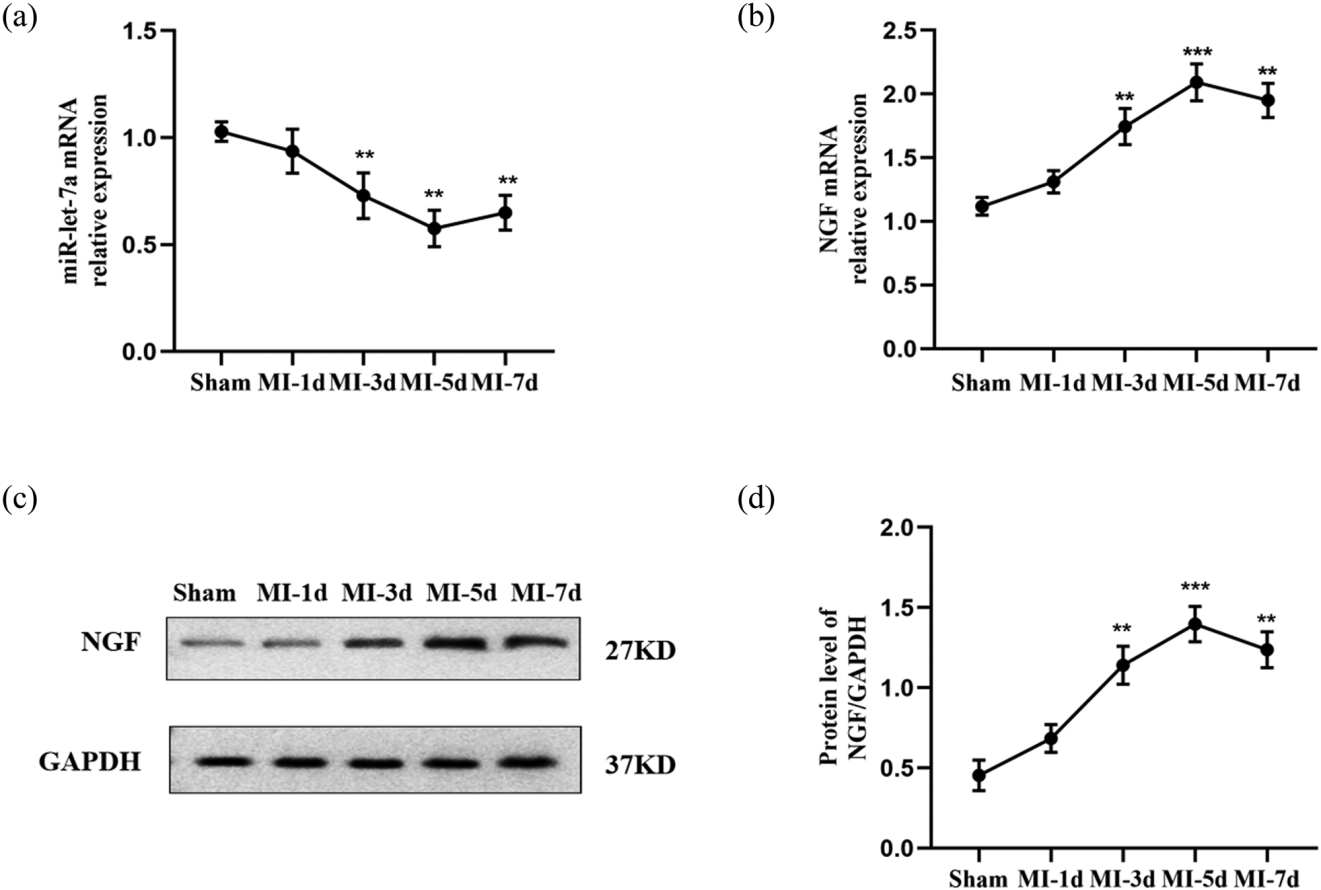
Expression of miR-let-7a and NGF in rat hearts after MI. (a and b) miR-let-7a and NGF mRNA expression were measured in the sham group and 1, 3, 5, and 7 days after MI groups by RT-qPCR. (c) Representative western blots and (d) densitometric quantification of NGF protein. Relative levels of NGF were quantified by normalizing to GAPDH levels. *n* = 5 per group. Data are presented as the mean ± SD. **p < 0.01 and ***p < 0.001 vs the sham group. miR, microRNA; NGF, nerve growth factor; MI, myocardial infarction; SD, standard deviation.

To determine whether miR-let-7a performs important functions in MI, its expression level was measured in the hearts of adult SD rats after MI. The results indicated that the expression of miR-let-7a in the infarcted border area began to downregulate at 1-day post-MI (*p* > 0.05, not significant) and significantly decreased from 3 to 7 days post-MI compared with that in the sham group (Figure 1a). Furthermore, the expression level of NGF mRNA was significantly upregulated from 3 to 7 days post-MI compared to that in the sham group (Figure 1b). Western blotting results confirmed that the change in NGF protein expression was similar to that of the mRNA (Figure 1c and d).

### 3.2 miR-let-7a inhibits the expression of NGF in macrophages

**Figure 2:**
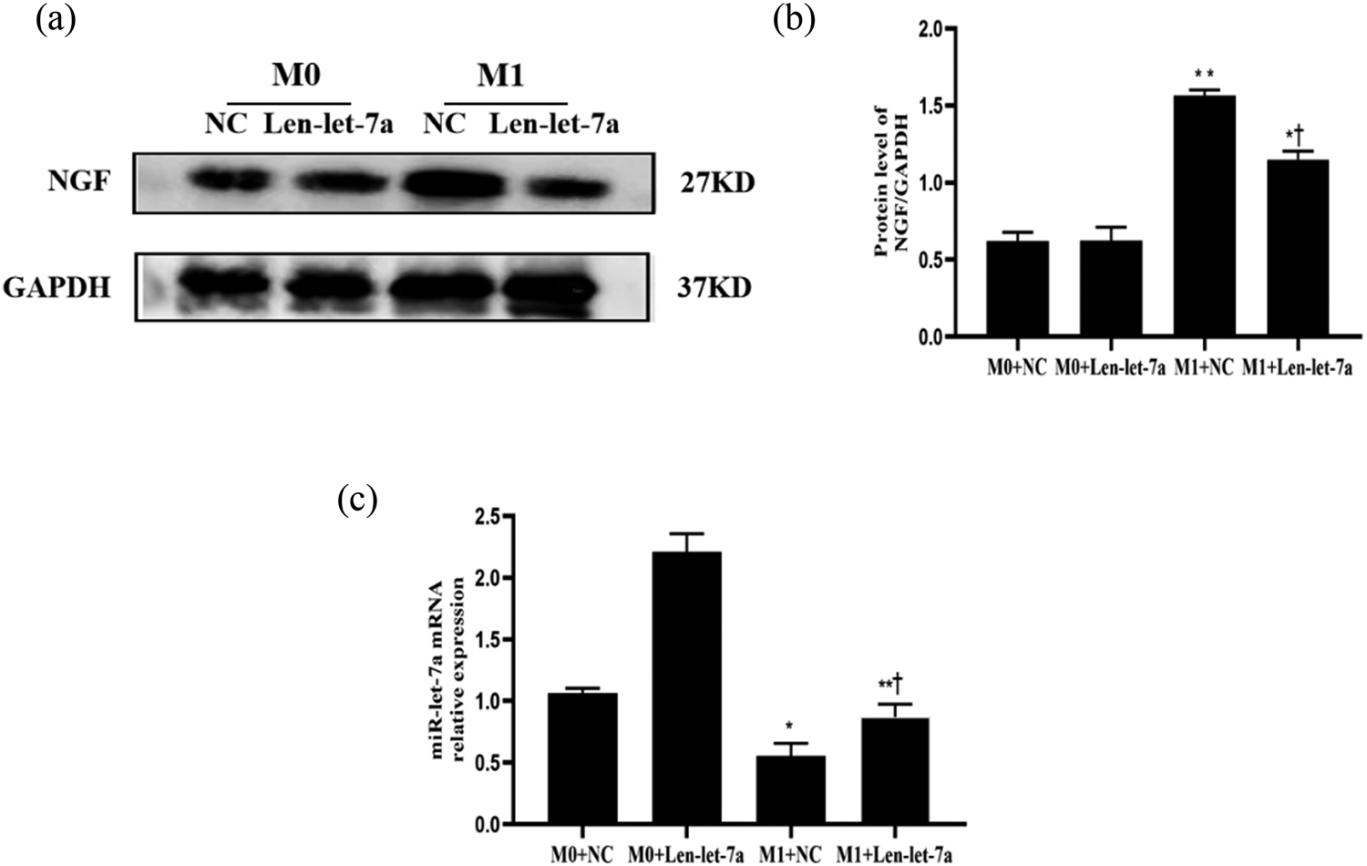
Verification of the relationship between miR-let-7a and NGF *in vitro*. (a) Expression of NGF in the M0 + NC, M0 + Len-let-7a, M1 + NC, and M1 + Len-let-7a groups as assessed via western blotting. (b) The blots were quantified using the protein expression level of GAPDH as the loading control. (c) Differences in expression of miR-let-7a in the M0 + NC, M0 + Len-let-7a, M1 + NC, and M1 + Len-let-7a groups as assessed by RT-qPCR. Data are presented as the mean ± SD. *p < 0.05 and **p < 0.01 vs the M0 group. ^†^ *p* < 0.05 vs M1 + NC. miR, microRNA; NGF, nerve growth factor; M0, M0 macrophages; M1, M1 macrophages; NC, negative control; Len, lentivirus; SD, standard deviation.

We aimed to clarify the expression of miR-let-7a and NGF in the M0 macrophages and M1 macrophages. M0 macrophages were transformed into M1 macrophages following induction with lipopolysaccharide (LPS) and gamma interferon (IFN-γ) (Figure A2). In M1 macrophages, the NGF concentration in the M1 + Len-let-7a group was significantly lower than that in the M1 + NC group (Figure 2). This result illustrates that miR-let-7a inhibits NGF expression in the macrophages.

### 3.3 NGF is a target of miR-let-7a

**Figure 3:**
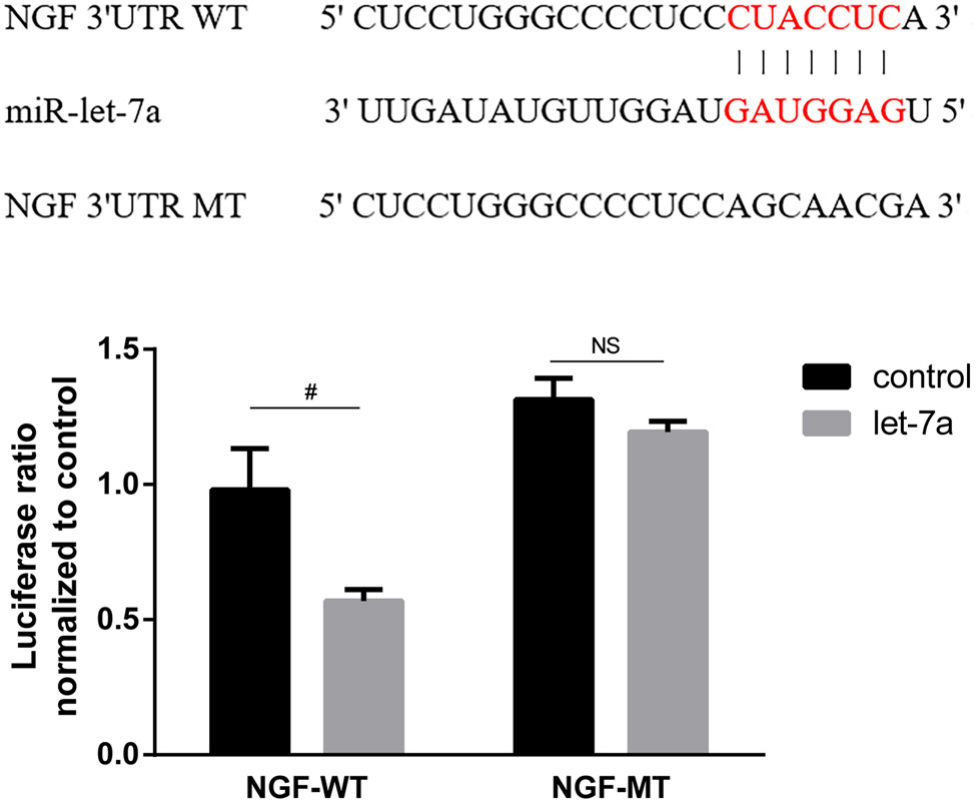
Prediction and identification of NGF as a target of miR-let-7a luminescence, mediated by a luciferase plasmid harboring the WT or MT NGF sequence, upon transfection with the miR-let-7a expression plasmid. A direct interaction between NGF and miR-let-7a was observed by dual luciferase reporter assay and statistical analysis. Data are presented as mean ± SD. ^#^ *p* < 0.05. miR, microRNA; NGF, nerve growth factor; UTR, untranslated region; WT, wild-type; MT, mutant.

To elucidate the molecular mechanism by which miR-let-7a affects sympathetic hyperinnervation post-MI, target genes of let-7a were predicted using TargetScan. Following the identification of NGF as a candidate target gene, a luciferase reporter assay was performed to confirm that NGF was directly regulated by miR-let-7a. The luciferase assay results showed that miR-let-7a suppressed the luciferase activity of the wild-type NGF 3′UTR more than that of the mutant NGF 3′UTR in 293T cells compared with the respective control, suggesting that NGF is a direct target gene of miR-let-7a (Figure 3).

### 3.4 Overexpression of miR-let-7a inhibits NGF expression and pro-inflammatory factor release after MI

**Figure 4:**
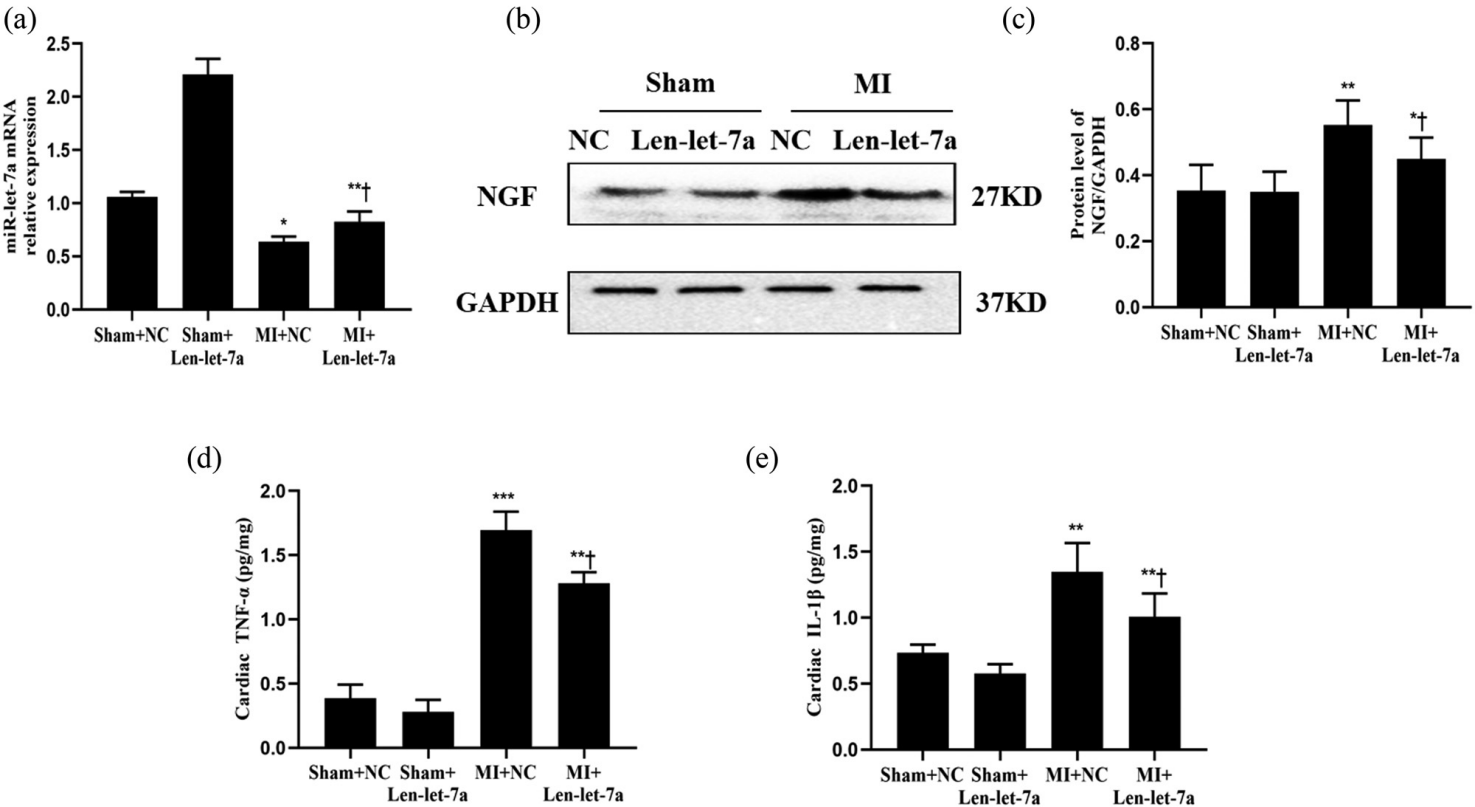
Overexpression of miR-let-7a inhibits the expression of NGF and inflammatory infiltration after MI. (a) Differences in expression of miR-let-7a among the sham + NC, sham + Len-let-7a, MI + NC, and MI + Len-let-7a groups as assessed by RT-qPCR. (b) Western blots and (c) quantification of the expression of NGF protein in the sham + NC, sham + Len-let-7a, MI + NC, and MI + Len-let-7a groups. The protein expression level of GAPDH was used as the loading control for quantification. The cytokine levels of IL-1β (e) and TNF-α (d) as measured by ELISA. *n* = 10 per group. Data are presented as the mean ± SD. *p < 0.05, **p < 0.01 and ***p < 0.001 vs the sham group. ^†^ *p* < 0.05 vs MI + NC. miR, microRNA; NGF, nerve growth factor; NC, negative control; Len, lentivirus; MI, myocardial infarction; SD, standard deviation.

Equal doses of miR-let-7a overexpression lentivirus or negative control virus were injected into the left ventricular wall of rats after MI. As previously mentioned, the MI group exhibited significantly decreased expression of miR-let-7a and increased expression of NGF compared to the respective expression levels in the sham group (Figure 4a–c). To further verify the regulatory relationship between miR-let-7a and NGF in the MI model, NGF, and miR-let-7a expression levels were compared in rats following the administration of Len-let-7a. The results revealed that the expression of NGF in the MI + Len-let-7a group was significantly lower than that in the MI + NC group, and the expression of miR-let-7a in the MI + Len-let-7a group was higher than that in the MI + NC group (Figure 4a–c). Previous studies have shown that sympathetic remodeling after MI is associated with inflammation [18]. The expression of inflammatory factors (TNF-α and IL-1β) in cardiac tissue was determined using ELISA. Compared to the sham group, the expression of inflammatory factors was significantly increased in the MI group (Figure 4d and e). However, the expression of inflammatory factors in the MI + Len-let-7a group was significantly lower than that in the MI + NC group (Figure 4d and e). In conclusion, miR-let-7a overexpression alleviated inflammatory infiltration after MI.

### 3.5 Overexpression of miR-let-7a reduces cardiac sympathetic remodeling after MI

**Figure 5:**
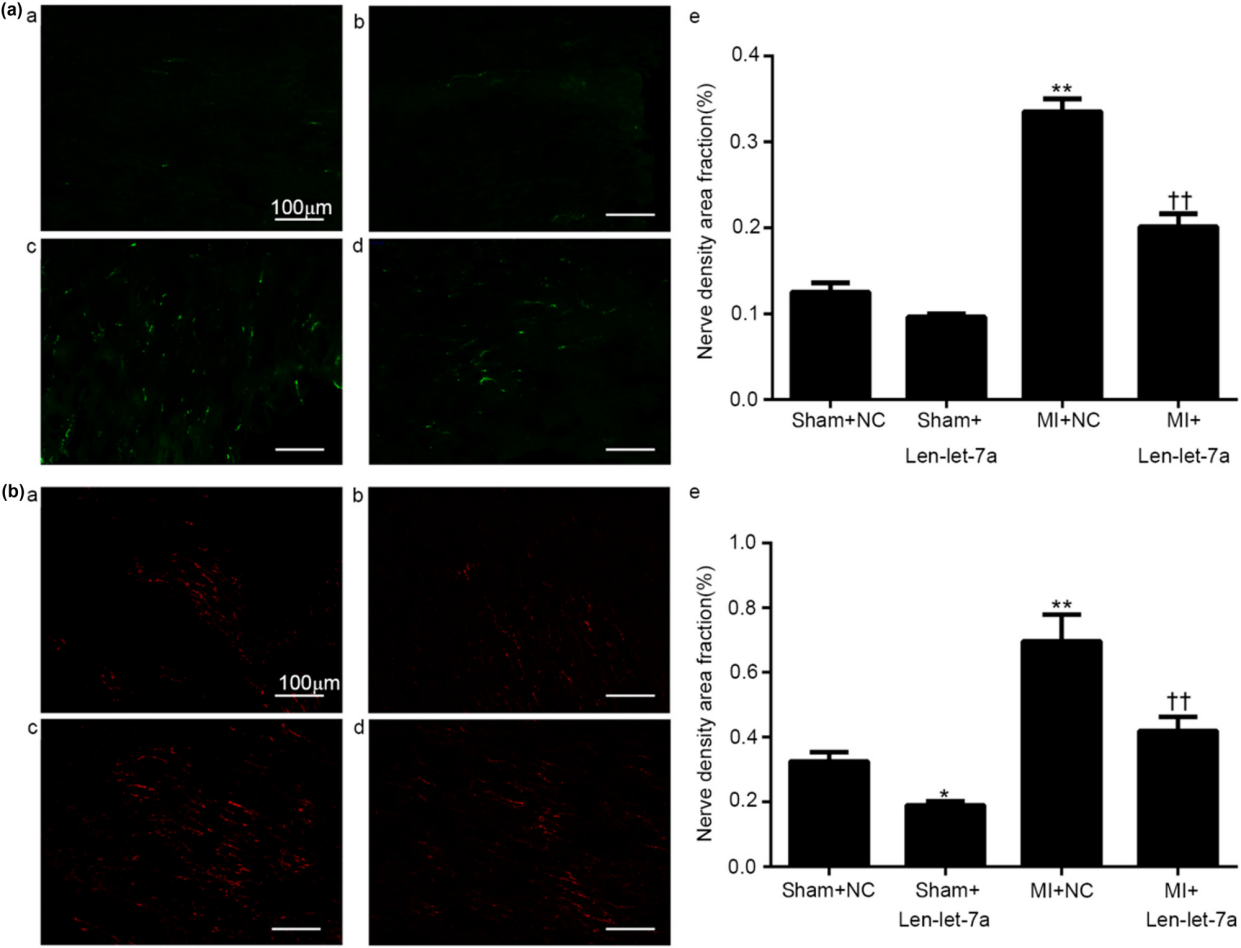
Overexpression of miR-let-7a attenuates sympathetic neural remodeling after MI. Staining of TH and GAP43 in rat hearts from the sham + NC, sham + Len-let-7a, MI + NC, and MI + Len-let-7a groups (magnification, ×200). (a) TH-positive nerve fibers from the infarcted border of the (a) sham + NC, (b) sham + Len-let-7a, (c) MI + NC, and (d) MI + Len-let-7a groups; (e) fraction (%) of TH-positive nerve density area. (b) GAP43-positive nerve fibers in the (a) sham + NC, (b) sham + Len-let-7a, (c) MI + NC and (d) MI + Len-let-7a groups; (e) fraction (%) of GAP43-positive nerve density area. *n* = 5 per group. Data are presented as the mean ± SD. **p < 0.01 vs the sham + NC group, ^††^ *p* < 0.01 vs the MI + NC group. TH, tyrosine hydroxylase; GAP43, growth-associated protein 43; NC, negative control; Len, lentivirus; MI, myocardial infarction; SD, standard deviation. **p < 0.01 vs Sham groups. ^††^ *p* < 0.01 vs MI + NC group.

As the MI group exhibited significantly decreased miR-let-7a expression compared to the sham group, the role of miR-let-7a in sympathetic remodeling after MI was further explored. Tyrosine hydroxylase (TH) (a marker of sympathetic nerves) and GAP43 (a marker of neuronal regeneration) levels were detected using immunofluorescence staining. The distribution of TH- and GAP43-immunostained nerve fibers was significantly higher in the MI + NC group than in the sham + NC group, while TH and GAP43 expression was significantly lower in the MI + Len-let-7a group than in the MI + NC group (Figure 5).

**Figure 6:**
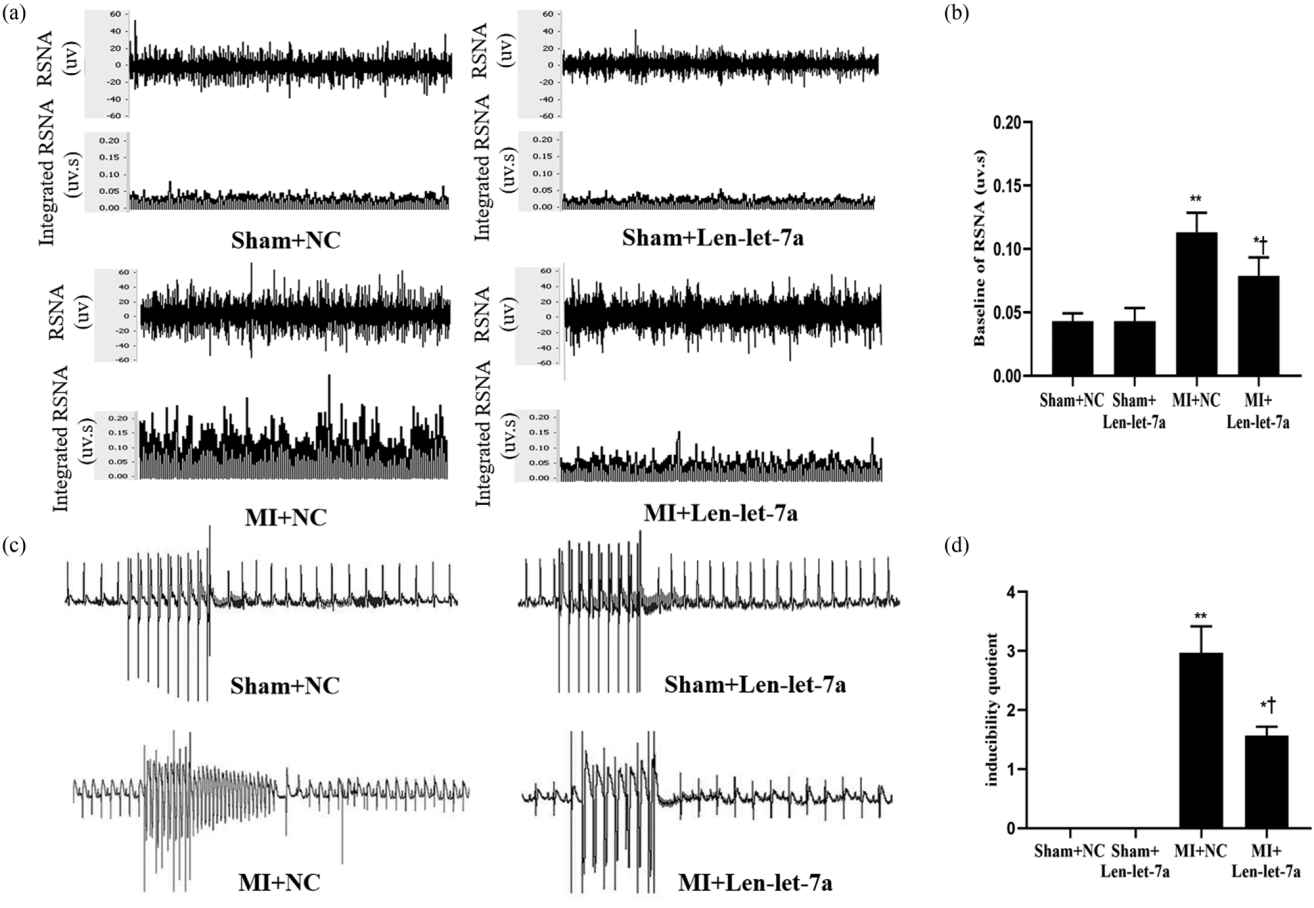
Effects of miR-let-7a on sympathetic nerve activity and programmed electrical stimulation at 7 days after MI. (a) Typical recordings from the left RSNA and integrated RSNA in the sham + NC, sham + Len-let-7a, MI + NC, and MI + Len-let-7a groups. (b) Baseline of RSNA. (c) Representative electrocardiogram of programmable electrical stimulation at 7 days after MI in the sham + NC, sham + Len-let-7a, MI + NC, and MI + Len-let-7a groups. (d) Comparison of arrhythmia scores between the four groups. *p < 0.05 and **p < 0.01 vs sham groups. ^†^ *p* < 0.05 vs MI + NC group.

To assess sympathetic nerve activity, the left RSNA, PES, and HRV were recorded. RSNA increased in the MI + NC and MI + Len-let-7a groups compared to that in the sham group (Figure 6a and b). RSNA was lower in the MI + Len-let-7a group than in the MI + NC group (Figure 6a and b), indicating the significance of miR-let-7a in sympathetic activation. PES indicated that rats were vulnerable to the induction of VA following MI. However, the VAs score decreased significantly in the MI + Len-let-7a group compared with that in the MI + NC group (Figure 6c and d), implying that overexpression of miR-let-7a had an inhibitory effect on the incidence of VAs after MI.

HRV analysis was performed to systematically evaluate the autonomic nerve control. The low-frequency (LF) component is correlated with sympathetic tone, whereas the high-frequency (HF) component is considered a marker of parasympathetic tone. The LF/HF ratio was used as an index of the interaction between sympathetic and parasympathetic activities. Compared with the MI + NC group, the MI + Len-let-7a group showed a downregulated LF component and LF/HF ratio (Table 2), indicating that the overexpression of miR-let-7a decreased sympathetic activity.

**Table 2:** HRV measurement data at the end of the study

Parameters	Sham		Ligation	
	Sham + NC	Sham + Len-let-7a	MI + NC	MI + Len-let-7a
No. of surviving rats	20	19	22	24
Survival rate (%)	100	95	73	80
HR, bpm	365 ± 9.7	378 ± 14.1	451 ± 14.6**	419 ± 10.9^*,†^
LF, nu	20.47 ± 2.09	20.65 ± 4.631	43.84 ± 6.13**	34.98 ± 5.52^*,†^
HF, nu	89.81 ± 3.43	88.20 ± 5.28	73.25 ± 6.21*	78.90 ± 6.19^*^
LF/HF	0.2279 ± 0.06	0.2341 ± 0.08	0.5984 ± 0.09**	0.4433 ± 0.08^*,†^

Data are presented as mean ± SD. HR, heart rate; HF, high frequency; Len, lentivirus; LF, low frequency; MI, myocardial infarction; NC, negative control. *p < 0.05 and **p < 0.01 vs sham groups. ^†^ *p* < 0.05 vs MI + NC group.

## 4 Discussion

The present study aimed to explore the role of miR-let-7a in the pathogenesis of sympathetic remodeling after MI to gain an improved understanding of the mechanism by which VA occurs and develops after MI. Several novel findings have been reported in this study. First, the results revealed that the expression of miR-let-7a significantly decreased after MI, and the trend in its expression was the reverse of that of NGF. This trend was more obvious in M1 macrophages. Second, miR-let-7a overexpression effectively inhibited the expression of NGF and the release of proinflammatory factors. Finally, the data suggested that the overexpression of miR-let-7a inhibited sympathetic remodeling and sympathetic nerve activity and reduced the incidence of VA after MI. Together, these findings indicate that miR-let-7a inhibits sympathetic remodeling and VA following MI by downregulating NGF expression. Therefore, miR-let-7a may be a potential therapeutic target for reducing the mortality associated with MI by inhibiting the occurrence of VA.

There have been numerous studies on miRNAs that suggest their use as markers in the assessment of various cardiovascular diseases soon. For example, in a clinical study of patients with heart failure, a large number of differentially expressed miRNAs were detected in blood [19]. In another study, through statistical analysis of 21 patients with coronary heart disease and 21 normal controls, it was found that the secretion of miR-381 in patients with coronary heart disease was significantly downregulated and endothelial cells were protected from inflammatory cells through CXC chemokine receptor 4 and the MAPK signaling pathway [20]. In addition, the high expression of miRNA-92a in patients with acute myocardial infarction (AMI) is beneficial for cardiac vascular regeneration and functional repair after AMI and promotes ventricular remodeling [21]. Thus, certain miRNAs may serve as novel biomarkers for simple and rapid diagnosis and accurate treatment of cardiovascular diseases and provide a new field of research for the development of new drugs. As one of the earliest miRNAs to be identified, miR-let-7a participates in pathophysiological processes including cell proliferation and differentiation, angiogenesis, and organ development through a variety of mechanisms [20]. Several studies have shown that miR-let-7a is closely associated with heart diseases, such as dilated cardiomyopathy [21], congenital heart disease in children [22], heart failure [23], coronary heart disease [24], and pulmonary hypertension [25]. Although miR-let-7a has been shown to play an important role in cardiovascular disease, the mechanism of miR-let-7a in arrhythmia requires further study. The present study found that the expression of miR-let-7a decreased after MI, while the expression of nerve remodeling-associated factors, such as TH and GAP43, significantly increased; the susceptibility to VA also increased. However, administration of a miR-let-7a-overexpressing lentivirus suppressed the expression of TH and GAP43 and decreased the susceptibility of rats to VA after MI. This indicates that miR-let-7a may serve as a potential biomarker for the simple and rapid diagnosis and treatment of VA after MI.

Macrophages are important innate immune cells of the body and have many functions, such as phagocytosis, antigen presentation, immune defense, and inflammatory regulation, and they participate in the initiation and resolution of inflammation. When the body experiences trauma, macrophages play a role in the immune response by secreting active cellular substances, including tumor necrosis factor, interleukins, and NGF, to promote wound repair [8]. Within 7 days of the occurrence of MI, a large number of monocytes gather in and near the infarcted area, where they differentiate into macrophages, induce inflammation, release NGF, inflammatory factors, angiogenic mediators, and other substances, remove necrotic substances, and promote angiogenesis and cardiac remodeling. Leor et al. [26] reported that in cases of acute MI, early administration of human-activated macrophages induced the continuous proliferation of myocardial fibroblasts, indicating that the macrophages contributed to ventricular remodeling, prevented further expansion of the infarction area, and effectively improved cardiac function. Our previous study found that in the early stage of MI, macrophages are the main cells that secrete NGF, and most of these macrophages are M1 macrophages, which are the main drivers of sympathetic remodeling after MI [27]. NGF has been studied as a potential biomarker by scientists worldwide and has been demonstrated to bind to the high-affinity receptor tropomyosin receptor kinase A and the low-affinity receptor p75-neurotrophin receptor [28]. Zhou et al. [29] demonstrated that NGF expression in the heart significantly increased 3 h after MI in dogs, and 1 week later, NGF expression in the non-infarcted area was significantly higher than that in the infarcted area. NGF expression after MI is closely associated with cardiac sympathetic nerve remodeling, and inhibition of NGF function affects the activation, regeneration, and remodeling of sympathetic nerves [9]. The results of the present study are consistent with the aforementioned results, indicating that NGF is an important contributor to sympathetic remodeling, the expression of NGF is upregulated after MI, and susceptibility to VA is also increased. A previous study revealed that controlling the expression of miR-let-7a promotes phenotypic changes in macrophages and affects the inflammatory response [30]. Another study found that miR-let-7a expression decreased in macrophages in the synovial fluid of patients with rheumatoid arthritis [31]. It was also revealed that anti-citrullinated protein antibodies (ACPAs) inhibit miR-let-7a in macrophages, while miR-let-7a targets high mobility group A2 to inhibit the activation of ACPA-induced macrophages, thus participating in the regulation of autoimmune disease. Therefore, in the present study, we investigated the regulatory relationship between miR-let-7a and NGF in a rat model of MI and M1 macrophages. In the rat model of MI, the expression of miR-let-7a decreased and the expression of NGF increased compared to that in the sham controls. Following local administration of miR-let-7a overexpression virus into the myocardium, NGF expression was significantly reduced. NGF expression was also downregulated following the transfection of the miR-let-7a overexpression virus into M1 macrophages, indicating that a negative regulatory relationship may exist between miR-let-7a and NGF. Therefore, we suggest that in M1 macrophages, the overexpression of miR-let-7a inhibits sympathetic remodeling after MI by downregulating NGF expression, which ultimately reduces the incidence of VA.

There are also some limitations in this study. First, we have not performed any histology around the infarct zone to observe whether there is any difference in fibrosis in the MI area induced by inflammation infiltration. Second, we have not examined cardiac functions to observe whether reduced inflammation infiltration with miR-let-7a overexpression protects the heart against MI injury. In the future, we will perform HE staining and electrocardiogram to further explore those issues.

## 5 Conclusions

To the best of our knowledge, the present study is the first to conduct an in-depth investigation of the regulatory effect of the miR-let-7a-macrophage-NGF axis on post-MI nerve remodeling and ventricular arrhythmia. Future studies are required to further explore the upstream factors that regulate miR-let-7a and the mechanism of sympathetic nerve remodeling. The findings of this study may provide novel insights for the development of new drugs and targeted clinical interventions for the treatment of arrhythmias following MI.
